# Biopolymeric Nanocomposites for Wastewater Remediation: An Overview on Recent Progress and Challenges

**DOI:** 10.3390/polym16020294

**Published:** 2024-01-21

**Authors:** Annu, Mona Mittal, Smriti Tripathi, Dong Kil Shin

**Affiliations:** 1 School of Mechanical Engineering, Yeungnam University, 280-Daehak-ro, Gyeongsan 38541, Republic of Korea; 2 Department of Applied Sciences (Chemistry), Galgotias College of Engineering and Technology, Greater Noida 201310, Uttar Pradesh, India

**Keywords:** biopolymers, chitosan, nanofiltration, membranes, adsorption, antibiotics, sustainable

## Abstract

Essential for human development, water is increasingly polluted by diverse anthropogenic activities, containing contaminants like organic dyes, acids, antibiotics, inorganic salts, and heavy metals. Conventional methods fall short, prompting the exploration of advanced, cost-effective remediation. Recent research focuses on sustainable adsorption, with nano-modifications enhancing adsorbent efficacy against persistent waterborne pollutants. This review delves into recent advancements (2020–2023) in sustainable biopolymeric nanocomposites, spotlighting the applications of biopolymers like chitosan in wastewater remediation, particularly as adsorbents and filtration membranes along with their mechanism. The advantages and drawbacks of various biopolymers have also been discussed along with their modification in synthesizing biopolymeric nanocomposites by combining the benefits of biodegradable polymers and nanomaterials for enhanced physiochemical and mechanical properties for their application in wastewater treatment. The important functions of biopolymeric nanocomposites by adsorbing, removing, and selectively targeting contaminants, contributing to the purification and sustainable management of water resources, have also been elaborated on. Furthermore, it outlines the reusability and current challenges for the further exploration of biopolymers in this burgeoning field for environmental applications.

## 1. Introduction

Globally, human beings are facing two basic challenges, namely, the dearth of clean water and its contamination. Water is a fundamental need to sustain life. Natural and anthropogenic activities produce large quantities of micropollutants in water [1]. Industrial development and advancements in agricultural techniques accelerate the accumulation of non-degradable pollutants in aquatic life [2]. Organic and inorganic impurities end up in lakes, rivers, or oceans, and the oxygen content in these is then affected [3]. The organic content immediately starts consuming oxygen in the water, resulting in oxygen deficiency, which in turn leads to the death of fish and other aquatic animals. This is due to the unnaturally high consumption of oxygen by pollutants [4]. If inorganic nutrients such as nitrogen and phosphorus are discharged into the water, they provide a food source for algae and plankton. This new biomass is organic matter, and when it decomposes, it consumes additional amounts of oxygen [4]. Small quantities of nutrients can create a large amount of biomass and result in substantial oxygen depletion and extensive damage to the aquatic system. The impact of effluents from the specified wastewater is contingent upon the characteristics of the receiving water [5]. Discharges containing organic matter may pose harm in certain contexts. Conversely, in other areas, the release of phosphorus and nitrogen could lead to significant environmental harm by fostering biological growth [6]. Thus, it is imperative that the issuance of discharge permits and the selection of purification methods aligns with the ecological requirements of the region [2]. The rapid surge in global population and widespread industrialization poses significant challenges in ensuring access to safe drinking water [7]. This pressing issue underscores the urgency for the exploration of effective and cost-efficient water treatment methods. Addressing this need is crucial for sustaining the well-being of communities and ecosystems in the face of escalating demands on freshwater resources [7,8].

Different techniques and methods are used to treat wastewater purposely to maintain the quality and quantity of water contaminated by natural or anthropogenic activities [9]. Non-futuristic approaches like unplanned industrialization and urbanization and the use of pesticides, synthetic fertilizers, and antibiotics or medical waste play a significant role in polluting water; thus, the availability of freshwater is still a challenge [10]. Antifouling is one of the critical problems for treating wastewater [11]. Conventionally, different modifications have been observed in polysulfone membranes by adding poly(2-acrylamido-2-methyl-1-propanesulfonic acid) and Cu_2_O for the ultrafiltration of proteins, BSA, and humic acid from the water by increasing the antifouling properties [12,13]. A water treatment process typically involves many important steps, which may vary in order and complexity, depending upon the kind of contamination present in water. For the elimination of contaminants, different physical, chemical, and biological methods are recommended. The conventional methods of water treatment involve the use of strong chemicals and organic media. The main steps of water treatment are coagulation and flocculation [14,15]. Coagulation implies the addition of coagulants like alum or ferric chloride to the water, which neutralizes the electrical charges of particles present in wastewater, and flocculation implies soft stirring to promote the formation of substantial particles that settle down easily [14,16]. The process is followed by sedimentation, filtration, disinfection, and pH adjustment. The introduction of membranes in water treatment upgraded the process by reducing its cost and making it an eco-friendly approach [17]. To fulfil the demands of fresh water, recycling and reusing contaminated water were adopted. The tertiary step in the water treatment process concentrates on the removal of floating organic contaminants with phosphorus and nitrogen [18]. Advance treatment includes advanced oxidation processes (AOPs), which use strong oxidation reactions to break down complex and tenacious pollutants, membrane bioreactors; use membrane filtration and constructed wetlands; and use wetlands to treat wastewater [19,20,21]. Out of the throng of wastewater treatment processes, adsorption is the most recommended process because of its comprehensibility, efficiency, regeneration capacity, and cost-effectiveness [22]. The degree of adsorption is calculated using suitable adsorbents for components; it is essential to elucidate the physical and chemical aspects of the adsorbent and the related mechanism [23]. Since the effectiveness of each technique depends on the specific characteristics of the wastewater and the targeted contaminants, the choice of method often involves a combination of techniques to achieve optimal results. Table 1 depicts the advantages and disadvantages of different techniques utilized for treating varying types of wastewaters.

Nanomaterials are practical and efficient solutions to get through major roadblocks in creating effective remedial technologies for wastewater treatment [24]. The large surface-to-volume ratio and numerous reactive sites of nanomaterials make them highly reactive toward quick and efficient removal of water pollutants [25]. Nanostructured adsorbents can be specifically designed to target pollutants and possess a substantial capability for addressing contaminated water [26,27]. There is also growing research focusing on the synthesis of biodegradable polymers for wastewater remediation. Natural polymers called biopolymers are either produced from sustainable natural resources or biosynthesized by living organisms [28]. Biopolymers are mostly composed of polysaccharides, and polypeptides. Biopolymers can be divided into three categories: nature-derived, chemically produced, and microbial biopolymers [28,29]. Renewability, biocompatibility, environmental compatibility, biodegradability, and antimicrobial activity are only a few of the impressive interrelated biological, physical, and chemical characteristics of biopolymers [29,30]. Reactive functional groups such as carbonyl, amide, carboxyl, and hydroxyl are present in the skeleton of biopolymers, which make them suitable for wastewater treatment [31]. However, the cost inefficiency of the synthesis and purification procedures is a significant problem that has been noticed throughout the scaling up of biopolymers [32]. Recently, there has been a surge in the scientific significance of biopolymer nanocomposites owing to their versatile applications in addressing environmental issues and remediation challenges [33]. Biopolymeric nanocomposites have been used to remove heavy metals, natural organic matter, dyes, antibiotics, and other water pollutants such as coagulants, adsorbents, flocculants, membranes, and photocatalytic agents [11,34]. Biopolymeric nanocomposites have enhanced physiochemical, thermophysical, and mechanical properties compared to nanomaterials and polymers [35]. Inorganic nanofillers such as metal and metal oxide nanoparticles, nanoclays, and carbon nanomaterials can be included in a biopolymer matrix to produce biopolymeric nanocomposites [36,37,38].

**Figure 1:**
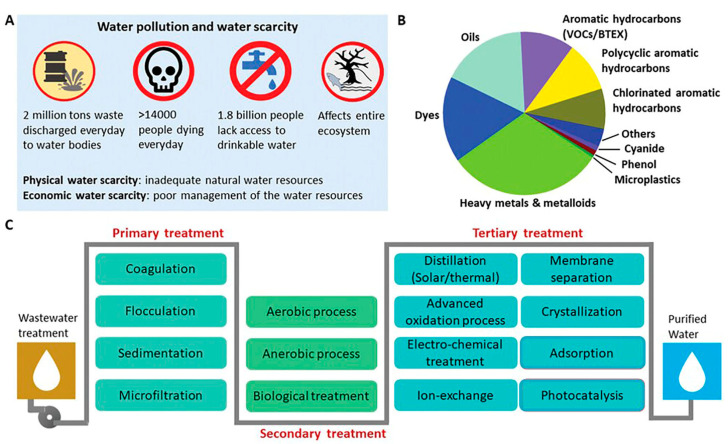
General illustration of polluted water and technologies available. (**A**) Global scarcity of water and pollution caused by (**B**) different pollutants, and (**C**) different stages of technologies for wastewater treatment [39].

The primary objective of this comprehensive review is to conduct a thorough analysis of the current status and progress in the field of biopolymeric nanocomposites, with a specific focus on their utilization for water remediation. This review explores production methods, properties, and applications of various biopolymeric nanocomposites, emphasizing particularly their role as filtration membranes and adsorbents in the context of wastewater treatment. Beginning with a brief introduction and discussion of the advantages associated with biopolymeric nanocomposites, this review then covers diverse synthesis methods, their properties, and recent advancements in applications and modifications in the composition of a biopolymeric nanocomposite. Furthermore, this paper openly discusses the functions, mechanism, reusability, limitations, and challenges of these materials, emphasizing their significant potential for further exploration and refinement in the field of water remediation. Moreover, this review article supports and advances the UN’s sustainable development goals, in particular, SDG 7 (Affordable and clean energy) and SDG 13 (Climate Action). Figure 1 shows a comprehensive overview of global water scarcity, the diverse array of pollutants impacting water quality, and stages of wastewater treatment—primary, secondary, and tertiary—aimed at removing inorganic, organic, and biological contaminants for effective water purification.

**Table 1:** Advantages and disadvantages of different wastewater remediation techniques.

Technique	Type of Wastewater	Advantages	Disadvantages	Refs.
Filtration	Pharmaceutical industry Fish processing	Simple and widely applicable Effective removal of suspended solids	Limited removal of contaminants Filter media can get clogged, require frequent maintenance	[40,41]
Coagulation	Domestic sewage Oil Surface water Algae-laden	Efficient removal of colloidal particles Enhances subsequent filtration processes	Formation of sludge imposes on proper disposal Requires careful control of coagulant dosage	[42]
Precipitation	Acidic decontamination of radioactive concrete Digested swine	Can reduce water hardness Effective for the removal of dissolved heavy metals	pH control is crucial for precipitation reactions Sludge production and disposal challenges	[43,44]
Adsorption	Urban Pharmaceutical Organic	High efficiency in removing organic pollutants Versatile with various adsorbent materials	Saturation of adsorption sites over time Regeneration of adsorbents can be complex	[45,46,47]
Flocculation	Pb (II)-polluted groundwater	Aggregation of particles for easier removal Enhanced sedimentation and filtration	Requires careful control of flocculant dosage Potential carryover of fine particles	[48]
Electrodialysis	N and P High-salt organic Carbocysteine	Selective removal of ions Continuous operation with minimal chemical usage	High energy consumption Scaling on membranes may occur	[49,50,51]
Membranes	Textile Microelectronic	Effective removal of particles, microorganisms, and ions Applicable for various contaminants	High operational and maintenance costs Membrane fouling can reduce efficiency	[52,53]
Ion exchange	Cu (II), Ni (II) Cu (II), Pb (II) Municipal Mining	Selective removal of specific ions Regeneration allows for extended use	Limited to ion-specific removal High regeneration chemical usage	[54,55,56,57]

## 2. Why Biopolymeric Nanocomposites?

In water remediation processes, biopolymeric nanocomposites serve several important functions and advantages. Firstly, biopolymeric nanocomposites are often derived from natural sources, making them environmentally friendly. Their use in water remediation aligns with sustainable practices, contributing to eco-friendly and green approaches for water treatment [58]. The adsorption capacity of biopolymeric nanocomposites enables them to effectively remove pollutants from water. Biopolymeric nanocomposites, due to their high surface area and functional groups, can adsorb or attract contaminants present in water. Contaminants adhere to the surface or interact with the nanocomposite’s structure, facilitating their separation from the water. This includes pollutants such as heavy metals, dyes, organic compounds, antibiotics, and other impurities [59]. Additionally, biopolymeric nanocomposites can act as filtration agents. They can be designed with specific properties to trap or filter out particulate matter, microorganisms, microplastics, or other undesirable components from water, contributing to improved water quality [60]. Some biopolymeric nanocomposites possess ion exchange capabilities. This means they can exchange ions with contaminants in water, effectively reducing the concentration of harmful substances [61,62]. Furthermore, a notable feature of certain biopolymeric nanocomposites is their ability to be regenerated and reused. After adsorbing contaminants, these nanocomposites can undergo a regeneration process, allowing them to maintain their adsorption capacity for multiple treatment cycles [63]. Interestingly, some biopolymeric nanocomposites can be tailored for selective binding to specific contaminants, such as antibiotics or other chemical compounds. This selectivity enhances their efficiency in targeting particular pollutants without affecting the overall composition of water [64,65]. In summary, biopolymeric nanocomposites play a pivotal role in water remediation by adsorbing, removing, and selectively targeting contaminants, contributing to the purification and sustainable management of water resources.

## 3. Biopolymeric Nanocomposites

Biopolymers are defined as degradable polymers derived from natural sources such as chitosan, alginate, pectin, lignin, starch, cellulose, etc., along with some biodegradable synthetic polymers such as polylactic acid, polyhydroxybutyrate, polyhydroxyalkanoates, etc., and play a vital role in the formation of biopolymeric nanocomposites [28,66,67,68,69,70]. Those derived from synthetic sources, however, are not renewable and do not entirely adhere to the notions of renewability and degradability. Biopolymer-based nanocomposites are also known as bionanocomposites by some researchers [71]. The unique structure, physiochemical characteristics, chemical stability, and high reactivity of biopolymers make them attractive candidates. The presence of functional groups on biopolymers facilitates the absorption of water pollutants, and hence, biopolymers are suitable for wastewater treatment. Polysaccharides are one of the biopolymers that are frequently used because of their eco-friendliness, biodegradability, nontoxicity, etc. Through physical and chemical interactions, they can also bind to various substances [28]. They are the perfect choice for water treatment because of their adsorption capabilities [72]. Due to the growing societal concern for the environment, environmentally friendly and sustainable concerns have a wide spectrum of appeal. As a result, materials are created according to their life cycle between extraction and disposal. In the cycle, it is also necessary to assess their negative effects on the environment. The utilization of renewable sources rather than synthetic ones to make environmentally friendly polymeric nanocomposites, known as biopolymeric nanocomposites, avoids the challenges associated with plastic waste. These materials are entirely renewable in terms of energy and biodegradable, in addition to being environmentally benign. As a result, these materials can be disposed of at the end of their useful lives without endangering the environment. Biopolymeric nanocomposites use inorganic nanoparticles as nanofillers distributed in an organic biopolymer matrix to combine the advantages of both [33]. Nanofillers are classified as zero-dimensional (0D), one-dimensional (1D), and two-dimensional (2D) nanofillers depending on their dimensions in the nanoscale region. Fillers with all dimensions less than 100 nm are referred to as 0D nanofillers. Similarly, fillers with one or two dimensions less than 100 nm are called 2D and 1D nanofillers, respectively [73,74]. The morphology of the produced biopolymeric nanocomposites, the size of the nanomaterials, and the class of the polymers are some parameters to categorize biopolymeric nanocomposites [75,76].

Among the array of biopolymers, chitosan stands out due to its notable antimicrobial attributes, biodegradability, and impressive gelation properties [77]. These qualities position it as an exemplary material with versatile applications, extending beyond wastewater treatment to various scientific and technological fields. Non-toxicity, biodegradability, antimicrobial, antioxidant, and biocompatibility properties are significant beneficial properties of chitosan, which make it a universal biopolymeric nanocomposite candidate [66]. The positively charged amino groups present on the surface of chitosan enable its interaction with negatively charged contaminants or ions. Due to this reason, the application of chitosan is not only confined to environmental applications but also emerges in different fields of science and technology. Biopolymers such as chitosan with its distinctive gel-forming capacity, particularly when coupled with nanoparticles like Ag ZnO, TiO_2_, etc., [73,74,78] in bionanocomposites or nanocomposites, hold great promise in food packaging [79], biomedical [78,80,81], and textile applications [82,83], in addition to environmental applications [84,85]. Accompanied by interesting characteristics, chitosan has some drawbacks as well including limited selectivity for certain contaminants.

Alginate, derived from seaweed, has gel-forming ability in the presence of divalent cations and therefore it has good affinity toward metal ions and organic pollutants. It is biocompatible and suitable for encapsulation. However, it lacks in maintaining mechanical strength, stability, and preventing disintegration in aggressive chemical environments, which can affect its performance. Similarly, all the biopolymers have some pros and cons for their utilization in wastewater treatment. Table 2 describes the advantageous characteristics and limitations of biopolymers in wastewater treatment.

## 4. Synthesis of Biopolymeric Nanocomposites

**Figure 2:**
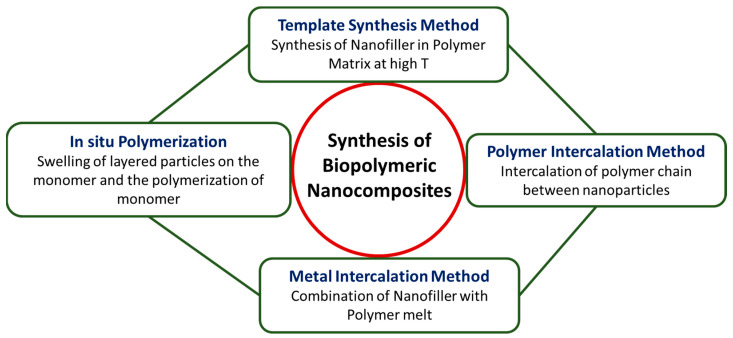
Representation of various synthesis methods of polymer nanocomposites.

Biopolymeric or polymeric nanocomposites, in general, have been synthesized using various methods, such as the template synthesis method, melt intercalation, polymer intercalation from a solution, and the in situ polymerization method as mentioned in Figure 2.

In the template method, filler material is synthesized in the presence of a polymer matrix at high temperatures. Consequently, the polymer facilitates the initiation and expansion of the inorganic host crystals while ensnaring them within its layers. Although it has the potential to produce exfoliated nanocomposites, filler aggregation cannot be neglected [123].

In the melt intercalation process, the high-molecular-weight polymer is heated to a high melting point and combined with the filler as the polymer melts. As a result, neither a solvent nor a chemical synthesis is required in this procedure. However, this procedure can be difficult for high-molecular-weight polymer chains in the filler interlayers owing to the thermodynamic and kinetic impacts on intercalation. Therefore, filler modification is necessary for exfoliating the polymer matrix under shear action [123].

In polymer intercalation using the solution method, the polymer is soluble in a solvent while the nanoparticles are dispersed in a solvent. In the aftermath, the polymer adheres to the delaminated sheets, with subsequent solvent evaporation. As the sheets reassemble during solvent evaporation, they entrap the polymer chains within their layers. This process results in the formation of a multilayered structure [124].

In the in situ intercalation method, the layered particle undergoes swelling in the monomer, initiating monomer polymerization thereafter. The resulting structure is considerably intercalated or exfoliated because of the monomer being present both inside and outside of the filler interlayers. This procedure results in the formation of stable nanocomposites [125,126]. To produce polymer nanocomposite materials, the selection of precursors, design, and synthetic techniques is crucial. Producing polymeric nanocomposites with specified properties involves a meticulous selection of monomers, fillers, and other composite materials, along with the application of distinct synthesis techniques. This highlights how important the design and synthesis processes are in the production of polymeric nanocomposites [127].

Chemical and mechanical methods are mostly efficient techniques for improving the dispersion of nanoparticles in polymeric nanocomposites. Enhancing the interaction or surface area between polymer matrices and nanoparticles is crucial in the design of polymeric nanocomposites [128]. The utilization of a surfactant is thought to be a useful method for enhancing the interaction between the organic phase of the polymer matrix and the inorganic phase of the nanoparticles. Several studies demonstrated the use of silane as a surfactant for inorganic phase surface modification and increasing their dispersion in the polymer matrix. The esterification process of hydrolyzed vinyltrimethoxysilane in an alcoholic solution was used to successfully silanize nanodiamonds [129]. Nanoparticles are compelled to disperse across the polymer matrix through agitation, one of the mechanical procedures. Ultrasonic or high-frequency sonication dispersion is also beneficial in this area since it offers more uniform dispersion as opposed to agitation approaches [130]. Nanoparticle aggregation problems can also be resolved via atomic layer deposition and plasma-assisted mechanochemistry [130]. There are various surfactants used for inorganic phase surface modification in polymer matrices. However, depending on the specific materials and applications, different surfactants may be employed. For the wastewater remediation process, the surfactants can either be cationic, for example, cetyltrimethylammonium bromide (CTAB) [131], used for the surface modification of negatively charged inorganic particles, or anionic (sodium dodecyl sulfate, sodium lauryl ether sulphate) [132,133] with vice versa surface modification, or non-ionic surfactants (Triton X-100, polyoxyethylene glycerol ester) [134], which do not possess a charged head, and are generally used for the dispersion of hydrophobic pollutants and oil droplets in wastewater. Additionally, triblock copolymers such as pluronic surfactants are used in wastewater treatment for the stabilization and dispersion of nanoparticles or colloids [135,136]. Furthermore, fluorinated surfactants, for example, perfluorooctanoic acid (PFOA), are also employed for the treatment of fluorinated compounds of industrial wastewater [137].

## 5. Properties of Polymeric and Biopolymeric Nanocomposites

**Figure 3:**
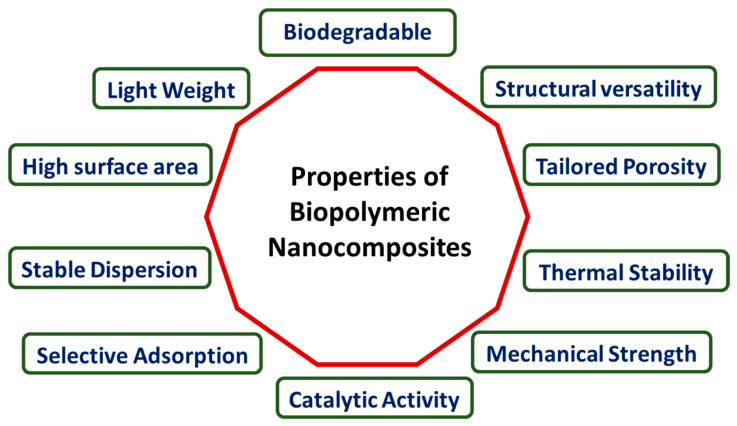
Properties of biopolymeric nanocomposites.

The advancement of suitable polymer nanocomposites has significantly augmented the advantageous attributes of polymers, potentially introducing a novel set of features for the resultant materials, as depicted in Figure 3. The extent of improvement, however, hinges on factors such as the nanomaterial’s shape, size, aspect ratio, dispersion state, and interfacial interactions with the polymer matrix. The enhancement of mechanical properties, including the tensile strength, modulus, or stiffness, is often a primary motive for incorporating nanoparticles into polymer matrices. Nonetheless, achieving even dispersion is crucial, as poor compatibility between polymer matrices and inorganic particles can lead to flaws that adversely affect the mechanical properties of polymer nanocomposites. Utilizing nanoparticles has proven effective in addressing the dimensional stability of neat polymers at elevated temperatures, attributed to their high thermal expansion coefficient, contributing to the overall improvement in thermal stability [71]. Polymer materials often have low electrical conductivity. Conductive polymeric nanocomposites are made possible by combining polymer matrices with conductive nanoparticles and are useful in electronic circuits. These goods exhibit not just electrical conductivity but also particular polymeric component characteristics, including flexibility and cheap production costs [126,138].

In another aspect, biopolymeric nanocomposites exhibit a range of properties that make them advantageous for various applications, particularly in wastewater treatment. Some key properties include the following: Enhanced mechanical strength—biopolymeric nanocomposites often display improved mechanical properties compared to their individual components. The addition of nanomaterials reinforces the structural integrity of the biopolymer, enhancing its overall strength. High surface area—the nanoscale features of these composites contribute to a large surface-to-volume ratio. This property increases the available surface area for interactions, making them effective in adsorption processes. Biodegradability—biopolymeric components, such as chitosan, are inherently biodegradable. When combined with nanomaterials, the resulting nanocomposites often maintain biodegradability, making them environmentally friendly. Tailored porosity—nanocomposites can be engineered to have specific porosity levels. This tunable porosity enhances their adsorption capacity, making them suitable for capturing pollutants in wastewater. Chemical stability—the combination of biopolymers and nanomaterials can lead to enhanced chemical stability, ensuring the composite remains robust in various environmental conditions. Selective adsorption—the presence of nanomaterials provides selective adsorption capabilities, allowing the nanocomposites to target specific pollutants or contaminants in wastewater. Thermal stability—the incorporation of nanomaterials often improves the thermal stability of biopolymeric nanocomposites, making them suitable for applications involving varying temperature conditions. Versatility—biopolymeric nanocomposites can be versatile in terms of composition and structure, allowing for customization based on specific wastewater treatment requirements. Understanding and leveraging these properties contribute to the effectiveness of biopolymeric nanocomposites in addressing challenges related to water pollution and wastewater treatment.

## 6. Applications of Biopolymeric Nanocomposites in Wastewater Remediation

One of the significant hurdles to achieving sustainability and an eco-friendly world is protecting the existing water resources. Less than 1% of the world’s water supply is considered clean, while the remaining water is polluted as per international standards [139]. Municipal wastewater, industrial waste, and agricultural practices are the main causes of water contamination. Among the diverse categories of pollutants, including organic acids, heavy metals, pesticides, fertilizers, dyes, phenolic compounds, halogenated chemicals, and microorganisms, it is noteworthy that certain examples within each category exhibit dual characteristics of toxicity and non-biodegradability [34,84,140,141,142,143,144]. The intake of contaminated water also contributes to several ailments, such as cancer, fever, diarrhea, nasal septum rupture, skin irritation, chills, ulcers, organ damage, headache, abdominal pain, appetite loss, and a lot more. To ensure that all living species have access to clean water, these pollutants must be removed. In this respect, multiple cutting-edge technologies for water purification have since been created.

Conventional wastewater treatment plants mitigate water pollution by eliminating organic and suspended solids. However, with evolving standards and treatment approaches, there is a growing emphasis on the removal of both hazardous substances and organic matter. The methods used to remove these pollutants from sewage can be divided into three groups: physical, biological, and mechanical. There are several methods for treating contaminated water, including filtration, coagulation, precipitation, adsorption, flocculation, electrodialysis, membrane technologies, and ion exchange. Each of these procedures has both advantages and disadvantages. For instance, the precipitation process produces waste, which must be treated before disposal. The ion exchangers are quickly contaminated, lowering their capability for exchanging ions. However, considerable amounts of non-recyclable waste are produced during the flocculation and coagulation processes. Electrodialysis has limited application owing to its high operating costs and energy needs, while photocatalytic techniques require a lengthy reaction time to be effective. Adsorption and membrane technologies have gained a lot of attention in the past few years for water treatment. These techniques are also lacking in several areas that need to be addressed to make them an inexpensive and suitable solution for industrial use [145]. Recently, biopolymeric nanocomposites have become popular as filtration membranes and adsorbents for wastewater treatment.

### 6.1. Biopolymeric Nanocomposites as Filtration Membranes

Biopolymer-nanocomposite-based filtration membranes leverage the unique properties of nanomaterials integrated into biopolymer matrices, offering enhanced filtration performance, improved mechanical strength, and heightened resistance to fouling. These membranes hold great promise for diverse applications in water purification, separation processes, and environmental remediation. Both academia and industry have paid significant attention to water filtration membranes for desalination, microbial treatment, and ion permeation. Membrane-based separation technologies represent a pinnacle in advanced separation methodologies, lauded for their simplicity, adaptability, and cost-effectiveness. Operating as selective barriers, membranes facilitate the passage of desired materials while detaining undesired substances on their surface. Offering a diverse array of separation techniques, including ultrafiltration, reverse osmosis, and nanofiltration, these membranes stand out for their energy efficiency by eliminating the need for phase change and exhibit exceptional selectivity in removing trace pollutants from water [146].

**Figure 4:**
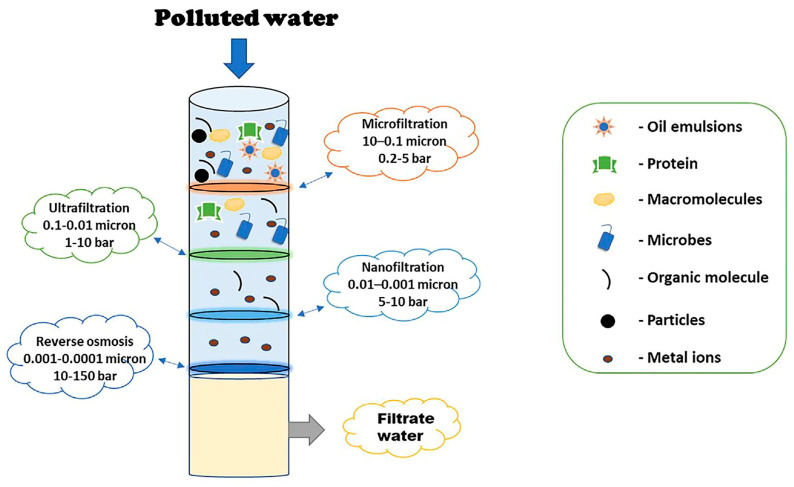
A schematic representing the capability of different membranes for treating wastewater [145].

Various membrane technologies cater to specific separation requirements, such as ultrafiltration, microfiltration, nanofiltration, forward and reverse osmosis, gas separation, membrane distillation, pervaporation, membrane bioreactors, and separation using liquid membranes, as represented in Figure 4. Reverse osmosis (RO) and nanofiltration membranes are especially extensively employed due to their high-water permeability, low-pressure requirements, and cost-effectiveness.

In the realm of wastewater treatment using adsorptive membranes, two fundamental approaches come into play: adsorption and rejection. When water-borne solutes encounter the membrane’s active layer, molecular sieving and filtration work collaboratively to reject solutes larger than the pore size. Simultaneously, smaller solutes penetrate the support layer, which acts as an adsorption microsphere. As these smaller solutes pass through the active layer, they form complexes, ultimately resulting in the production of filtered water through the absorptive membrane. This multifaceted process showcases the versatility and efficacy of membrane-based separation technologies in addressing the challenges of water purification and pollutant removal [34,84].

Inorganic membranes offer strong mechanical, structural, and thermal resistance. Despite their great selectivity, they are not suited for a wide range of applications due to their limited permeability. On the other hand, polymeric membranes have a low cost, easy manufacturing, excellent flexibility, chemical stability, and mechanical strength. Polyvinyl alcohol (PVA), polyether sulfone (PES), polyamide (PA), polyethylene (PE), polyvinylidene fluoride (PVDF), polyvinyl chloride (PVC), polypropylene (PP), polyacrylonitrile (PAN), polyimide (PI), chitosan, and alginate [147] are among the materials used to make polymeric membranes. Poly(ethylene glycol) (PEG), a non-biodegradable yet non-toxic polymer, undergoes modification with poly(vinylidene fluoride-co-hexafluoropropylene), incorporating methoxy PEG. This modification results in a material achieving a 99% rejection of humic acid and displaying robust antifouling properties, albeit with environmental considerations due to its non-biodegradable nature [28]. There are various reports on the use of polymeric membranes as filtration membranes for removing pollutants. However, there are several issues with the thermal and mechanical characteristics of current polymeric membranes used in water treatment techniques [148]. Filtration can be improved by using nanocomposite membranes, mixtures of nanofillers, and polymeric membranes. Nanofillers, which comprise metal/metal oxide nanoparticles and carbon-based nanoparticles, have received a lot of interest. It has been demonstrated that biopolymeric nanocomposites may successfully remove a variety of contaminants from wastewater to acceptable levels. Polymeric adsorptive membranes are powerful water pollution remediation solutions. Various kinds of persistent and developing chemical contaminants that are resistant to current approaches can be removed from wastewater using cellulosic and other polymeric membranes. Biopolymeric membranes captivate attention through their compelling integration of adsorption and filtration mechanisms. These membranes not only enhance membrane permeability but also elevate selectivity, rejection rates, and adsorption capacity. In addition to these performance improvements, the utilization of adsorptive membranes effectively tackles fouling issues, contributes to reduced operational costs, and enhances the reusability of the adsorbent. This harmonious synergy of adsorption and filtration mechanisms positions biopolymeric membranes as a promising avenue for advanced separation technologies. Several studies have been conducted to investigate the efficacy of biopolymeric nanocomposites in the removal of antibiotics from water sources. These investigations reveal the pivotal role played by these nanocomposites in the removal process.

In a study, Moradi et al. developed a high-performance thin-film composite nanofiltration membrane for antibiotic removal in pharmaceutical wastewater treatment. Utilizing furosemide-modified chitosan (CS@FS) composite-assisted pectin (PC) functionalization, the polyethersulfone (PES) nanofiltration membrane is enhanced in physicochemical characteristics, such as a smoother membrane surface and a reduced water contact angle. The optimized TFC membrane, TFC-0.5, achieves a 47.8 L/m^2 ^h pure water flux, 94.2% flux recovery ratio, and 5.8% irreversible fouling ratio. Additionally, the CS@FS-co-PC nanofiltration membranes excel in pharmaceutical wastewater treatment, with a 92.0% ± 1.1 COD removal efficiency, 56.1 ± 1.0% TDS removal, and whole turbidity removal. The membrane’s high antibiotic rejection and antifouling abilities make it promising for pharmaceutical wastewater treatment applications [149]. Similarly, Gopal et al. have developed a nanocomposite for the removal of antibiotics from water, employing clay-nanosheets supported with an Fe-Cu nanocomposite. This innovative approach involves immobilizing the composite in a biodegradable chitosan-coated alginate–carboxymethyl chitosan matrix, forming nanocomposite beads suitable for use in column reactors. The study demonstrates effective ciprofloxacin (CIPRO) removal, achieving approximately 90% under optimal conditions in the batch mode, with a maximum removal capacity of 485.58 mg/g according to the Langmuir isotherm. Additionally, the nanocomposite’s performance is assessed against various environmental factors, including salts (NaCl and CaCl_2_) and micro-contaminants (humic acid and polyethylene), providing valuable insights into its robustness. The research explores reaction parameters in column reactors, such as the flow rate, initial CIPRO concentration, and bed height. The study also evaluates the residual toxicity of the composite beads, confirming a substantial reduction in toxic effects on environmentally relevant algae (Chlorella sp. and Scenedesmus obliquus) [150]. Palacio et al. tackled the global challenge of antibiotic contaminants in water, focusing on nalidixic acid removal using two cationic polymers: poly[(4-vinylbenzyl) trimethylammonium chloride] and N-alkylated chitosan. The removal processes are governed by electrostatic interactions, π–π interactions, and hydrogen bonding, as revealed by their effectiveness under varying conditions, with distinct removal rates—75.0% at pH 9 for poly[(4-vinylbenzyl) trimethylammonium chloride] and 65.0% at pH 7 for alkylated N-chitosan [151]. Valizadeh et al. innovatively tackled tetracycline (TC) antibiotic pollution by introducing a zinc ferrite/chitosan–curdlan magnetic composite. This environmentally friendly adsorbent proved to be highly efficient in TC removal, with optimal conditions at pH 6 and a composite dosage of 0.65 g/L. The adsorption process adhered to pseudo-first-order kinetics and Langmuir isotherm models, revealing a maximum adsorption capacity of 371.42 mg/g at 328 K. A thermodynamic analysis suggested a spontaneous endothermic result and adsorption. The magnetic composite demonstrated easy separation, regeneration capability, and consistent stability over successive cycles, and was a cost-effective solution for removing pharmaceutical pollutants from water [152].

**Figure 5:**
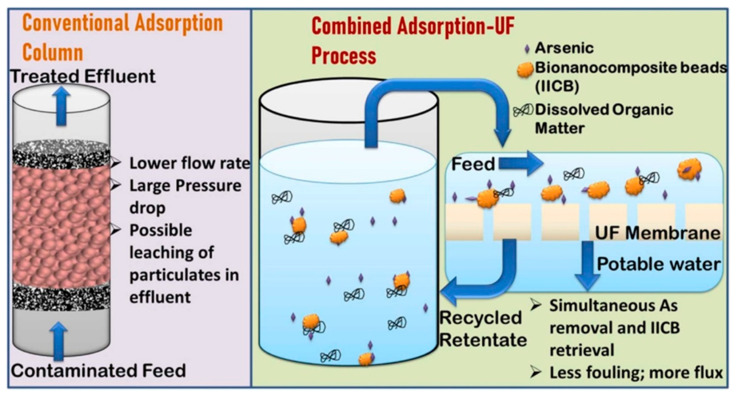
Schematic representation of conventional treatment of contaminated water with adsorption (**left**) and with ultrafiltration membranes (**right**) by using iron oxyhydroxide chitosan beads (IICBs) as the biopolymeric-chitosan-based bionanocomposite [153].

**Figure 6:**
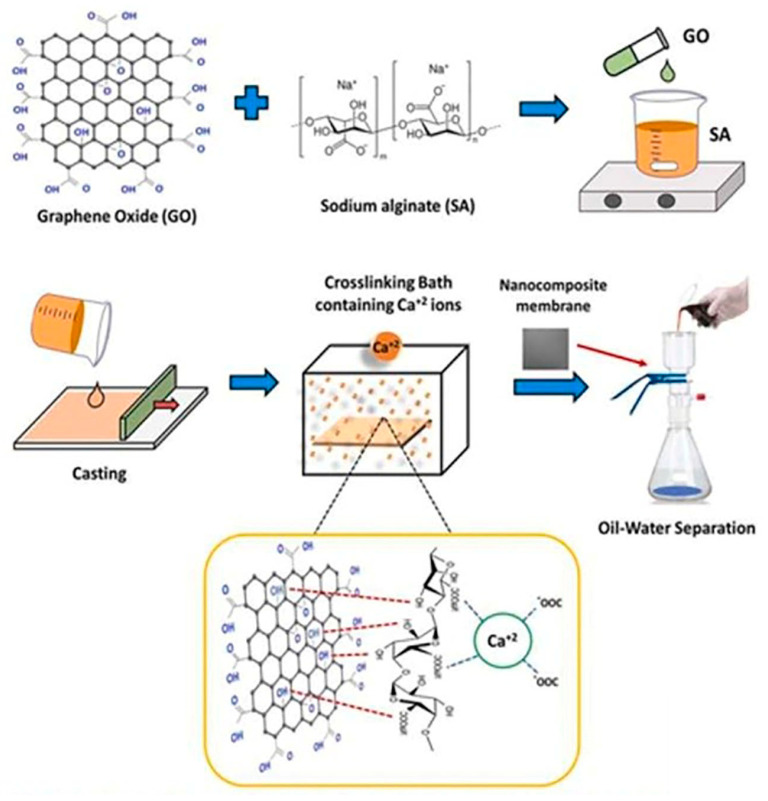
Schematic representation of oil–water separation by using alginate-GO-based nanocomposite membranes [154].

Rawat et al. have recently developed chitosan-based beads by using an iron oxyhydroxide metal nanocomposite for the ultrafiltration of contaminated water and removing arsenic. Their study revealed that a dose of 2 g/L of IICBs can remove arsenic to <10 µg/L permissible limits (Figure 5) [153]. Similarly, an alginate-based nanocomposite has been developed by Ehsan et al. in association with a graphene oxide (GO) carbon network for the separation of oil from water, as depicted in Figure 6 [154]. The utilization of biopolymeric materials, combined with nanotechnology, presents a promising avenue for addressing the environmental concern of antibiotic contamination in water.

#### Mechanism

**Figure 7:**
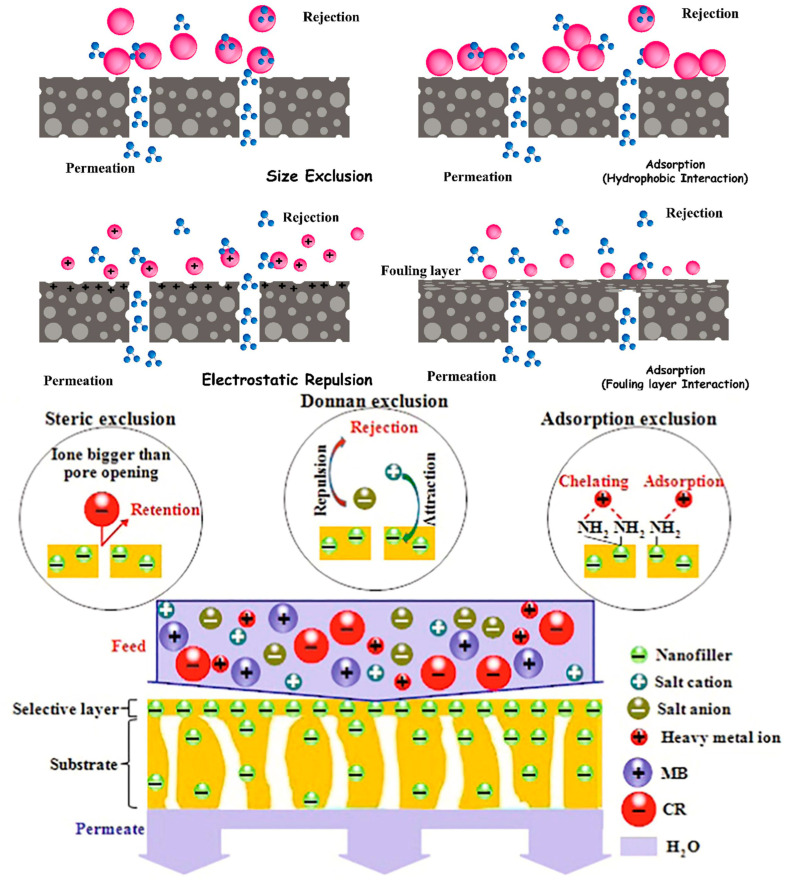
Mechanistic approach of membrane separation and rejection [58] of inorganic salt, heavy metal ions, and organic dyes [155].

The size exclusion mechanism operates by permitting molecules with dimensions smaller than the pore size to traverse the membrane, while larger species are impeded. Membranes featuring an accumulation of surface electric charge repel species carrying the same surface charges, facilitating the passage of neutral species as shown in Figure 7. The performance of membrane separation processes is significantly influenced by the physical and chemical interactions between chemicals and the membrane [58].

For instance, hydrophobic pollutants can engage in interactions with hydrophobic membrane surfaces through hydrophobic interactions, leading to the adsorption and retention of these species on the solid membrane. Conversely, the formation of biofilm on the membrane enhances the hydrophilicity of the surface, resulting in the rejection of hydrophobic pollutant species [58]. The mechanism of the separation of the rejection of pollutants such as inorganic salts, organic dyes, and heavy metal ions by using the biopolymeric nanocomposite membranes for nanofiltration is depicted in Figure 7 [155].

**Figure 8:**
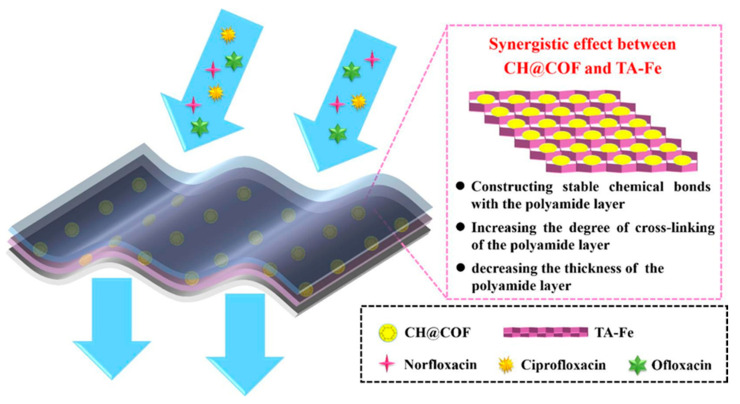
Pictorial representation of a thin-film composite nanofiltration membrane composed of chitosan hydrogel covalent organic framework interlayered with tannic acid-Fe^3+^ to remove norfloxacin, ciprofloxacin, and ofloxacin antibiotics from water [156].

The exceptional nanofiltration performance and stability of the thin-film composite nanofiltration membrane composed of a chitosan hydrogel covalent organic framework interlayered with tannic acid-Fe^3+^ involve the establishment of stable chemical bonding interaction between the substrate and polyamide layer and an increase in the degree of cross-linking within the polyamide layer, along with reduced thickness. The water permeability reached 16.17 L m^−2^ h^−1^ bar^−1^, marking a substantial increase to 185% compared to the TFC-control membrane’s 8.74 L m^−2^ h^−1^ bar^−1^. Furthermore, the membrane exhibited high rejections for norfloxacin (94.89%), ciprofloxacin (99.07%), and ofloxacin (99.10%). Notably, the flux recovery rate was impressive at 98.32% (alginate) and 97.99% (BSA), indicating remarkable antifouling performance (Figure 8) [156].

In recent years, the escalating global need for lithium resources has been fueled by the rapid expansion of the new energy sector. Regarding this, Zhang et al. have designed nanofiltration membranes, specifically with a positive charge, by utilizing modified chitosan as hydroxypropyltrimethyl ammonium chloride chitosan (HACC). The study revealed decreased thickness and increased hydrophilicity due to the interfacial polymerization process with HACC. Moreover, pore size remained unchanged, while the incorporation of the quaternary ammonium group in HACC significantly enhanced the antibacterial efficacy of the nanofiltration membranes. The optimized nanofiltration membrane, NF-HACC-0.3, significantly improved the separation factor and doubled the flux compared to the original membrane. This innovative approach of modified biopolymeric membranes shows high-performance capabilities in effectively separating magnesium ions (Mg^2+^) and lithium ions (Li^+^), and therefore serves as a valuable solution for the extraction as well as recovery of lithium resources from brine, addressing the growing demand in a sustainable manner [157]. Table 3 represents several biopolymer nanocomposite membranes that have been employed to filter out various types of water contaminants, along with treatment technology and their advantages.

### 6.2. Biopolymeric Nanocomposites as Adsorbents

For many years, biopolymers by themselves were utilized in the water purification process. Heavy metals, oil spills, and other particulates are successfully removed from wastewater using biopolymers. Furthermore, biopolymers and their derivatives can adsorb or capture heavy metals and have stronger adsorbing and chelating effects. The main contributors to water contamination are dyes, which are used in a variety of sectors, including printing, textiles, and painting. Most dyes that are released into water are poisonous and may have an adverse effect on photosynthetic activity by lowering sunlight penetration, which would therefore have an adverse effect on aquatic as well as human life. Therefore, it is crucial to get rid of these harmful dyes and save the environment. A variety of methods, including physical, chemical, and biological techniques, are employed for this purpose. The most popular physiochemical technique for achieving this goal is adsorption. Recently, MXene has been incorporated with chitosan/lignosulfonate nanospheres for removing heavy metals, viz., Cu(II), Co(II), Ni(II), Pb(II), and Cr(VI), from wastewater [170].

Biopolymers have been used extensively to eliminate harmful dyes and heavy toxic metal ions from aqueous solutions owing to their biodegradability, biocompatibility, and presence of multiple functional groups. However, their low thermal stability, poor mechanical properties, and small surface area limit their applications.

**Figure 9:**
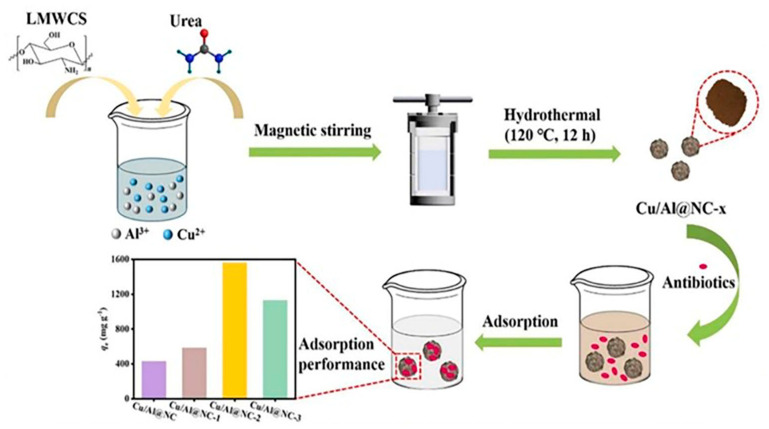
Schematic illustration of formation of low-molecular-weight chitosan (LMWCS)-Cu/Al with nitrogen-doped carbon microspheres, with hydrothermal method, as an excellent high-performance adsorbent for oxytetracycline antibiotic removal [177].

**Figure 10:**
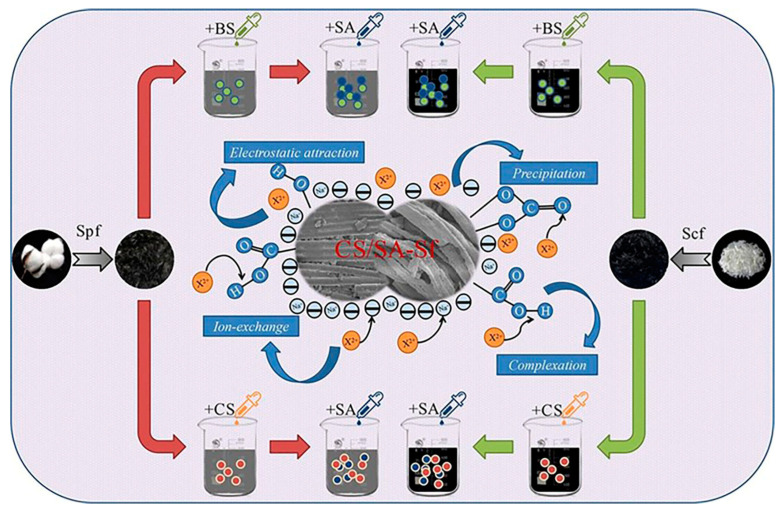
Schematic illustration of the preparation of semi-carbonized plant fiber (Spf) and chemical fiber (Scf) using dodecyl dimethyl betaine (BS) and chitosan (CS) as modifiers to enhance Sfs. Sodium alginate (SA) served as the composite modifier to further modify BS-Sf and CS-Sf (dodecyl dimethyl betaine and chitosan-modified semi-carbonized fibers), resulting in the preparation of BS/SA-Sf and CS/SA-Sf (sodium-alginate-composite-modified BS-Sf and CS-Sf) to remove Zn(II), Pb(II), and Cd(II) heavy metal ions from polluted water [178].

**Figure 11:**
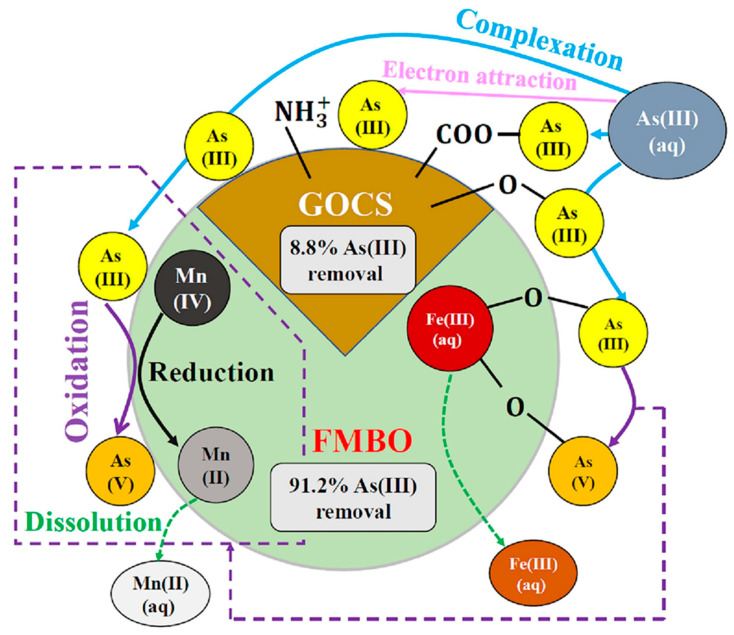
Schematic illustration of mechanism of removal of As (III) by using binary-doped Fe-Mn with chitosan-GO granular adsorbent [179].

GO/polyamidoamine nanocomposites have been investigated for the adsorption of Pb, Cu, Mn, and Cd heavy metal ions [171]. Magnetite nanoparticles were used to modify GO nanosheets before covalently attaching a dithiocarbamate-terminated highly branched polyamidoamine dendrimer to their surface. This study utilizes ultrasound-assisted magnetic solid-phase extraction for concentrating Ni(II), Cr(III), Cu(II), Pb(II), and Cd(II) to demonstrate their sorbent efficacy [172]. Hayati et al. demonstrated that the PAMAM/CNT nanocomposite is a super-adsorbent capable of absorbing unusually large amounts of heavy metals from single- and binary-component liquid phases [173]. ZnO nanoparticles were immobilized on the chitosan/silica hybrid to form an effective chitosan/silica/ZnO nanocomposite, which was used to remove methylene blue (MB) from wastewater using an adsorption process with a 293.3 mg/g adsorption capacity [85]. Similarly, a stable chitosan-TiO_2_ nanocomposite (CTNC) was synthesized for the quantitative and selective elimination of Rose Bengal dye from industrial wastewater with a 79.365 mg/g adsorption capacity [174]. A polyamidoamine dendrimer was successfully mounted on titania nanoparticles to create a novel nanohybrid with encapsulation potential for phenol removal from industrial wastewater [175]. Paleos and coworkers produced and characterized a variety of poly(propylene imine) dendrimers functionalized with extended aliphatic chains. These dendrimers have been shown to encapsulate polycyclic aromatic hydrocarbons from water down to the few-ppb level [176]. Huang et al. recently introduced carbon microspheres as an outstanding adsorbent by utilizing a chitosan biopolymer as depicted in Figure 9. A series of Cu/Al-doped nitrogen-containing carbon microspheres (Cu/Al@NC-x, x = 1, 2, 3) were then synthesized via a facile one-pot hydrothermal strategy. In experimental batch adsorption studies, these microspheres exhibited exceptional efficacy in removing oxytetracycline contaminants, contributing to water quality improvement. The subsequent thermodynamic analysis revealed a spontaneous endothermic process for Cu/Al@NC-2 adsorbing oxytetracycline (ΔH° > 0, ΔG° < 0). Notably, even after five adsorption cycles, Cu/Al@NC-2 maintained an excellent 92.25% removal efficiency for oxytetracycline [177]. Similarly, chitosan- and alginate-modified carbonized fibers have been recently developed by Li et al. to remove Zn(II), Pb(II), and Cd(II) heavy metal ions at pH = 6 and an optimized 30 °C temperature with a 0.1 mol/L ionic strength for maximum adsorption (Figure 10) [178]. Shan et al. delineated the mechanism for As(III) removal, emphasizing chemisorption as the predominant process. An Fe/Mn-doped chitosan-GO granular adsorbent facilitates the adsorption of most As(III) through inner-sphere complexation, specifically with Fe-O groups associated with ferrihydrite and goethite. This process coincides with the oxidation of As(III) to As(V), catalyzed by O_2_ and MnO_2_, followed by complexation with Fe-O groups. Additionally, a minor fraction of As is adsorbed through complexation with oxygen-containing functional groups, such as -OH and single -COOH, present in the chitosan-GO-based nanocomposite (Figure 11) [179]. Similarly, Zheng et al. has developed the composite nanofiber membrane based on the modified chitosan as carboxymethyl chitosan with a synthetic biodegradable polymer, polyvinyl alcohol, PVA, and GO by using the electrospinning method for the adsorption of heavy metal ions (Ni^2+^, Cu^2+^, Ag^+^, and Pb^2+^). The study shows the reduction in the nanofiber diameter and increased crystallinity through the addition of GO with improved intermolecular hydrogen bonding with the polymeric matrix. The improved adsorption capacity of the biopolymeric membrane for Ni^2+^, Cu^2+^, Ag^+^, and Pb^2+^ was observed at 262.1, 237.9, 319.3, and 413.6 mg/g, respectively [180]. Similar results have been observed by Thakur et al. with more than a 90% removal efficiency [181]. Chitosan and dialdehyde cellulose have also been explored for the removal of heavy metal ions using a Schiff base reaction and followed by the graft copolymerization of acrylic acid [182].

**Figure 12:**
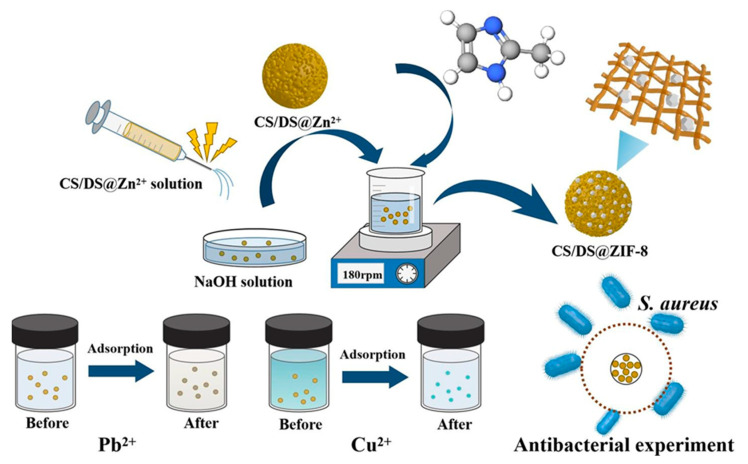
Schematic illustration of silica-doped chitosan with zeolite imidazolate framework (ZIF-8) composite microsphere for Pb^2+^ and Cu^2+^ heavy metal ion removal with significant antibacterial activity [183].

Li et al. formulated a bifunctional composite microsphere adsorbent, CS/DS@ZIF-8, resulting from the combination of a zeolite imidazolate framework (ZIF-8) with chitosan microspheres doped with silica (CS/DS), utilizing the electrospraying method. Characterization analyses indicated a superior crystallinity, increased specific surface area, diverse distribution of pore size, heightened thermal stability. Adsorption studies revealed that CS/DS@ZIF-8 adhered to the Langmuir model as well as the pseudo-second-order kinetics model, displaying maximal capacities of 340.94 mg/g for Pb^2+^ and 308.27 mg/g for Cu^2+^. These results demonstrated sustained adsorption rates of 81.3% for Pb^2+^ and 72.9% for Cu^2+^ over five cycles. This innovative microsphere effectively addresses both chemical and biological pollutants for water remediation (Figure 12) [183]. Wang et al. utilized advanced techniques, specifically freeze–drying and 3D printing, to fabricate a chitosan/hydroxyapatite, CS/HAP, composite adsorbent and a series of monolithic polylactic acid (PLA), PLA@CS/HAP, filters. This innovative method imparted distinctive macroscopic microchannel structures to the monolithic PLA@CS/HAP filters, significantly enhancing their Cu^2+^ removal capacity. The study revealed that the adsorption process aligned with Freundlich and pseudo-second-order models, suggesting a multi-layer adsorption with chemisorption characteristics for the CS/HAP composite adsorbent. Cu^2+^ reusability experiments demonstrated the resilience of PLA@CS/HAP filters, maintaining consistent Cu^2+^ removal capacity over five consecutive adsorption–desorption cycles, with a significant removal efficiency of 97.17% [184].

In another study, Ghiorghita et al. utilized a chitosan biopolymer, revealing the potential of ultra-lightweight thiourea–chitosan (CSTU) aerogels. These aerogels, with low densities (0.0021–0.0103 g/cm^3^) and high specific surface areas (416.64–447.26 m^2^/g), excelled in swiftly removing heavy metal ions. CSTU aerogels demonstrated impressive recycling stability (up to 80% removal efficiency after five cycles) and potent antimicrobial properties against bacterial strains. These findings underscore the CSTU aerogels’ potential in wastewater treatment and circular economy practices through biological decontamination [185]. Aspartame is a low-calorie artificial sweetener that has faced controversy and concerns due to potential health risks associated with its consumption. Khan et al. developed a green hydrogel nanocomposite, GTBCH, via free-radical polymerization for efficient removal of aspartame from wastewater. The robust adsorption capacity (392.04 mg g^−1^), as determined using the Langmuir model, can be ascribed to the enhanced interactions between AS and GTBCH. Their diffusion studies revealed aspartame uptake occurring through surface adsorption, liquid film, and intraparticle diffusion mechanisms, respectively [186]. In another study, Kebria et al. investigated the efficacy of a chitosan/polyethyleneimine composite xerogel for removing perfluorobutanesulfonic acid (PFBS) from aqueous solutions via static adsorption. The study covered a wide concentration range (ppb to ppm), revealing a maximum PFBS adsorption capacity of 305 mg/g within 24 h. Chemical characterization indicated electrostatic interactions and hydrogen bond formation between the xerogels’ amine groups and PFBS molecules; findings were confirmed using molecular dynamics simulations. This research offers a viable solution for PFBS removal, highlighting the composite xerogel’s potential in water treatment [187]. Basirun et al. synthesized a polymeric hydrogel, [HIMP][TS], through the functionalization of thiosalicylate-based ionic liquids, and integrated it into polyvinyl alcohol (PVA)–alginate beads for solid biomaterial support. The study focused on an effective treatment method for the removal of toxic manganese (Mn) heavy metal from industrial wastewater, employing an adsorption-based approach with an alginate adsorbent, incorporating the functionalized thiosalicylate-based ionic liquid [188]. Several innovative composite materials have been explored to tailor their mechanical characteristics and surface area, crucial for augmenting adsorption capacity, as outlined in Table 4.

## 7. Reusability of Biopolymeric Nanocomposites

Biopolymeric nanocomposites, a blend of natural polymers and nano-dimensional particles, are composed of eco-friendly components, proven to be remarkably useful in treating wastewater. Biopolymeric nanocomposites have crucial reusability. Their reusable nature means that after being used once to remove contaminants from water, they can undergo regeneration or be reintroduced into the treatment process multiple times [63]. This eco-friendly approach showcases the potential of biopolymeric nanocomposites as valuable tools in addressing water pollution challenges.

**Figure 13:**
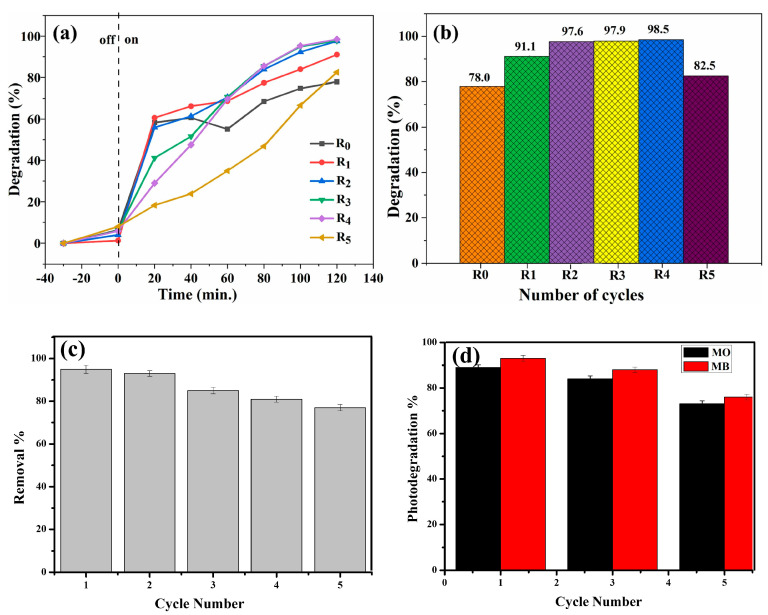
Reusability of biopolymeric nanocomposites: (**a**) chitosan-Fe_3_O_4_ nanocomposite for photocatalytic ability and (**b**) photodegradation of methylene blue dye for 120 min UV irradiation [203], and chitosan-based ternary nanocomposite with TiO_2_ and Ag nanoparticles on cellulose fabric (**c**) for removal of Cu (II) ions and (**d**) for photodegradation of methyl orange and methylene blue dye [204].

Reusability acts as a gauge for determining the stability of photocatalysts within environmental remediation systems. In another study, Salehi et al. demonstrated the elimination of organophosphorus pesticides, viz., chlorpyrifos and diazinon, from an aquatic region by using a MOF-based biopolymeric nanocomposite as a nanoadsorbent hydrogel. The adsorbent hydrogel was composed of xanthan gum, acrylamide, HKUST-1 as MOF material, and Fe_3_O_4_ magnetic nanoparticles. The reusability and cost-effective stability or sustainability of the fabricated hydrogel were best after four repeated cycles [202]. Similarly, Sudarmono et al. have evaluated the reusability of a chitosan-Fe_3_O_4_ nanoparticle (4:1)-based biopolymeric nanocomposite for the photodegradation of methylene blue. This study revealed high stability and reusability, in a respective 4:1 ratio, up to five cycles with an initial increase in photocatalytic degradation ability (13%) with a simultaneous decrease in the mass of the nanocomposite, which subsequently acts as a photocatalyst by 40%, as shown in Figure 13a,b [203].

Recently, Rehan et al. have developed a chitosan-based ternary biopolymeric nanocomposite with TiO_2_ and Ag nanoparticles, which were further deposited on cellulose fabric to evaluate the wastewater treatment efficacy in terms of removing methyl orange and methyl blue dye contaminants and Cu (II) ions from polluted water. Since the effectiveness of biopolymeric nanocomposites lies in their capacity to capture and eliminate pollutants from wastewater, their reusable feature enhances the sustainability of the treatment process, contributing to both environmental and economic advantages. Therefore, to demonstrate the stability and practical application of the cellulose-fabric-deposited chitosan nanocomposite, it was evaluated for reusability. In this regard, disodium ethylenediamine tetraacetate (Na_2_EDTA) was used as an eluent for the desorption test with the implication of five repeated cycles, and the test revealed a very reduced amount of loss (19%) in the adsorption ability of the nanocomposite fabric with the removal percentage of Cu (II) ions decreasing from 95% to 77% as depicted in Figure 13c. The reason for this decrement is due to the decrease in the number of actives responsible for the metal ion removal. Similarly, the stability and reusability of the nanocomposite cellulose fabric have been evaluated for the degradation of methyl orange and methylene blue organic dyes. The results revealed a 73% and 76% photocatalytic degradation of methyl orange and methylene blue dye, respectively, after five repeated cycles, as depicted in Figure 13d [204]. The main reasons for the decrease in the degradation activity are (i) a reduced mass after washing and drying, (ii) blockage of pores and active sites due to the accumulation of intermediate particles, and (iii) adherence of dye molecules to the surface of the photocatalyst after the fifth cycle [203].

## 8. Limitations and Challenges

Biopolymeric nanocomposites, while holding great promise for wastewater remediation, do have certain limitations that need to be considered. One of the primary challenges is the adaptation of biopolymeric nanocomposites for large-scale industrial applications. Ensuring scalability of synthesis methods and their integration into existing wastewater treatment systems is a complex task. Some biopolymeric nanocomposites may exhibit specificity in adsorption, limiting their effectiveness to certain types of contaminants. Ensuring broad-spectrum applicability requires addressing the specificity of adsorption. While some biopolymeric nanocomposites can be regenerated and reused, the efficiency of regeneration processes is either low or may vary. Enhancing the regeneration efficiency is crucial for maximizing the lifespan of these materials. Moreover, the cost of the production and implementation of biopolymeric nanocomposites can be a limiting factor. Ensuring cost-effectiveness compared to alternative treatment methods is essential for widespread adoption. The durability and stability of biopolymeric nanocomposites under different environmental conditions need thorough consideration. Long-term stability and resistance to degradation are crucial for sustained performance. The synthesis of certain biopolymeric nanocomposites may involve intricate processes. Simplifying and optimizing synthesis methods are essential for reducing complexity and enhancing efficiency. While selectivity can be an advantage, it may also limit the applicability of biopolymeric nanocomposites to specific types of contaminants. Achieving a balance between selectivity and versatility is a key challenge. The lack of standardized protocols for the synthesis and application of biopolymeric nanocomposites can hinder widespread adoption. Establishing standardized procedures is important for ensuring consistency and reliability. The availability of modified or functionalized biopolymeric nanocomposites tailored for specific contaminants may be limited. Expanding the range of available materials is crucial for addressing diverse water quality challenges. The environmental impact of synthesis processes for biopolymeric nanocomposites needs consideration. Ensuring that these processes align with sustainable and eco-friendly practices is essential.

## 9. Conclusions and Future Perspective

In conclusion, the synthesis and application of biopolymeric nanocomposites represent a promising avenue for advancing wastewater remediation strategies. Despite the significant advantages offered by these materials, including eco-friendliness, high adsorption capacity, filtration capabilities, ion exchange properties, and selective binding, several challenges and limitations must be addressed to fully realize their potential. The primary hurdle lies in adapting biopolymeric nanocomposites for large-scale industrial applications, requiring scalable synthesis methods and integration into existing wastewater treatment systems. Specificity in adsorption, regeneration efficiency, production costs, and the lack of standardized protocols further underscore the need for comprehensive research and development in this field.

To overcome challenges, future research efforts should prioritize enhancing the scalability of synthesis methods to facilitate seamless integration into industrial processes. Developing efficient regeneration protocols is imperative to prolong the lifespan of these materials and maximize their reusability. Additionally, optimizing production processes and exploring cost-effective alternatives are essential to ensure the economic viability of biopolymeric nanocomposites in comparison to conventional treatment methods. The balance between selectivity and versatility is crucial, and future studies should aim to strike this equilibrium, broadening the applicability of biopolymeric nanocomposites to diverse contaminants without sacrificing specificity. Standardized protocols for synthesis and application are paramount to ensure consistency and reliability across different studies, fostering widespread adoption and comparability of results. Looking ahead, the exploration of modified or functionalized biopolymeric nanocomposites tailored for specific contaminants should be a priority. Expanding the range of available materials will contribute to addressing diverse water quality challenges, catering to the unique characteristics of different pollutants. Moreover, a concerted effort toward sustainable and eco-friendly synthesis processes is necessary to align with global environmental goals and ensure the long-term viability of biopolymeric nanocomposites in water remediation.

In summary, this review provides an in-depth analysis of the current state of biopolymeric nanocomposites in wastewater remediation, offering insights into their advantages, functions, limitations, and challenges. Future perspectives outlined herein aim to guide and inspire further research endeavors, ultimately contributing to the evolution of and improvement in biopolymeric nanocomposites for sustainable and effective water treatment solutions.

## Figures and Tables

**Table 2:** Advantages and limitations of biopolymers in wastewater remediation.

Biopolymers	Advantages	Limitations	Refs.
Chitosan	High surface area and porosity High cationic charge density enables effective adsorption of anionic contaminants Biodegradable and environmentally friendly Versatile in various forms (powder, beads, membranes) for diverse wastewater applications	Limited stability in acidic conditions, impacting its performance in low-pH environments Relatively high production cost compared to some other biopolymers Regeneration for reuse can be challenging and may affect adsorption efficiency	[86,87,88,89,90]
Cellulose	Abundant and renewable, derived from plant sources Chemically modifiable for enhanced adsorption properties High surface area and porosity contribute to effective pollutant removal	Limited solubility in common solvents, affecting its processability May require chemical modification to tailor adsorption characteristics Production processes may involve energy-intensive treatments	[91,92,93,94,95]
Starch	Abundant, renewable, and cost-effective Chemically modifiable to enhance adsorption capacity Biodegradable and environmentally friendly	Relatively low mechanical strength in its native form Requires processing to improve stability and functionality Limited in applications requiring high-temperature stability	[96,97]
Alginate	Gel-forming properties in the presence of divalent cations Good affinity for metal ions and certain organic pollutants Biocompatible and suitable for encapsulation applications	Limited mechanical strength, which can affect its performance in certain applications Challenges in maintaining stability and preventing disintegration in aggressive chemical environments Possibility of cation exchange with divalent cations in water, leading to gel breakdown	[98,99,100,101]
Xanthan gum	High viscosity and excellent water-holding capacity Anionic nature facilitates interaction with cationic contaminants Rheological properties make it suitable for gel formation	High viscosity, which may hinder its dispersion and mixing in certain wastewater treatment processes Susceptibility to microbial degradation, affecting its long-term stability Limited adsorption capacity for certain types of contaminants compared to other biopolymers	[102,103,104]
Lignin	High aromatic content and complex structure Adsorption capacity for various pollutants due to functional groups Renewable and abundant, contributing to sustainability	Complex and heterogeneous structure, making it challenging to control and optimize for specific applications Limited solubility in water, which can impact its effectiveness in certain wastewater treatment scenarios The presence of impurities in lignin from various sources may affect its performance and reliability	[105,106,107]
Pectin	Biodegradable and environment-friendly Effective for the removal of specific pollutants from wastewater Structural feasibility for chemical modification to enhance adsorption	Limited biodegradability in certain wastewater treatment conditions, potentially leading to persistence in the environment Challenging processing while converting pectin into effective adsorbent forms Specific adsorption capabilities for certain pollutants	[108,109]
Carrageenan	Sulfated polysaccharide derived from red seaweed High binding affinity for metal ions and dyes Gel-forming properties enhance encapsulation of contaminants	Limited adsorption capacity for certain heavy metals The cost of production can be higher compared to other biopolymers May exhibit variability in performance based on carrageenan subtype	[108,110]
Pullulan	Water-soluble polysaccharide produced by yeast Forms inclusion complexes with various pollutants Biodegradable and suitable for controlled-release applications	Limited applicability to specific pollutants Relatively higher production costs Susceptible to microbial degradation under certain conditions	[111,112,113]
Cyclodextrin	Cyclic oligosaccharides with a hydrophobic core and hydrophilic exterior Forms host–guest inclusion complexes with organic pollutants Enhances solubility and bioavailability of certain contaminants	Limited adsorption capacity for larger molecules Higher cost compared to some other biopolymers Release of captured pollutants may require additional processes	[114,115,116]
Polylactic acid (PLA)	Biodegradable and eco-friendly Chemically modified PLA exhibits improved adsorption of pollutants Versatility in pollutant removal	Processing challenges for adsorbent forms High implementation costs Adsorbing specific pollutants may vary, requiring consideration of targeted contaminants	[117,118,119]
Polyvinyl alcohol (PVA)	Biodegradable and eco-friendly Adaptable for various forms, such as films, fibers, and gels Allows for chemical modification to tailor its properties	Biodegradation of PVA is influenced by specific environmental conditions, and complete degradation may require extended periods Incomplete degradation of PVA in wastewater treatment systems may lead to the accumulation of residuals, raising concerns about long-term environmental impact	[120,121,122]

**Table 3:** Recent studies on wastewater remediation by several biopolymeric nanocomposites as filtration membranes.

Nanocomposite	Type of Membrane	Pollutant	Flux Recovery Ratio/Rate	Advantages	Application	Refs.
Chitosan–iron oxyhydroxide beads	Ultrafiltration	Arsenic	-	Removal of toxic arsenic, reduction in fouling by 32 ± 2%	Portable drinking water	[153]
Alginate-GO	Nanocomposite	Oil	>88%	93.26% oil removal efficiency, good antifouling with 90% protein rejection rate	Oil–water separation	[154]
Chitosan-Fe_3_O_4_-SiO_2_	Nanofiltration	Na_2_SO_4_, MgSO_4_, NaCl MgCl_2_, Pb^2+^, Cu^2+^, Cd^2+^, dyes (MB, CR, RB5)	Water flux: 70.6 L m^–2^ h^−1^	High performance, high efficiency of heavy metal ion removal (98%), high rate of desalination, high retention of anionic dyes (BR5 and CR; ~98.2%)	Wastewater treatment	[155]
Chitosan-CNT	Nanofiltration	Brackish water	Water flux: 80.26 L/m^2^·h	95.5% salt rejection at 40 °C, remarkable water flux	Safe drinking water	[158]
Chitosan-PLA-Ag nanowires	Nanofibrous	*E. coli* and *S. aureus* bacteria	Ag leach out: 0.003 ppm, 36 h	Excellent antibacterial activity and removal of heavy ion contaminants	Potable drinking water	[159]
Chitosan	Ultrafiltration	Organic matter, inorganic salt	95%	Enhanced separation efficiencies, antifouling, and hydrophilicity, and reduced pore size	High-quality drinking water	[160]
Chitosan-GO	Nanocomposite	Bathroom greywater	Permeation: 23.43 kg/m^2^ h at 4 bars	High greywater treatment efficiency, improved porosity and water flux permeation, non-detectable pathogen inhibition	Reuse in non-potable application	[161]
Chitosan-Mil-125Ti nanoparticles	Nanofiltration	Organic dye, antibiotic, NaCl, Na_2_SO_4,_ and heavy metal	98% in bovine serum albumin (BSA) filtration	Enhanced performance for antifouling and high separation efficiency	Performance improvement in polyethersulfone (PES) membranes	[162]
Chitosan-MoS_2_-GO	Nanocomposite	Organic matter (dye, humic acid)	5.1 L m^−2^ h^−1^ bar^−1^	High porosity, 95–100% color removal, fast kinetics per filtration cycle, 100% (1 ppm) total organic content (TOC) removal	Separation and catalytic degradation of methyl orange organic dye	[163]
Chitosan-aminopropylsilane-GO	Nanocomposite	Pb (II) ion, C.I. Reactive Blue 50 and Green 19	>90%, water flux: 123.8 L/m^2^ h	98% BSA rejection, high removal efficiency (82%, Pb(II); 90.5%, Reactive Blue 50; and 98.5%, Reactive Green 19), and good antifouling properties	Filtration and separation	[164]
Chitosan-benzalkonium chloride-CNT	Ultrafiltration	BSA	Water flux: 88 (2 bars) to 138 L/m^2^ h (4 bars)	Increased porosity, minimized biofouling, decreased hydrophilicity, increased BSA rejection	Wastewater treatment	[165]
Alginate-PVA-GO	Nanofiltration	Lanasol blue 3R	88.7%	Improved permeability, porosity, and antifouling ability, >83% dye rejection	Water purification	[166]
Cellulose nanocrystal	Nanocomposite	Natural organic matter (humic acid, sodium alginate, BSA)	93.6% total fouling resistance	Increased performance of polyethersulfone membrane, improved antifouling ability and cleaning efficiency	Water treatment	[167]
Cellulose–chitosan-biomass-activated carbon nanoparticles	Molecularly imprinted membrane	Tetracycline antibiotic	-	High biodegradability, adsorption, and separation performance, 15.99 mg g^−1^ adsorption capacity, 4.91 perm-selectivity factor	Pollutant separation	[168]
N-phthaloylchitosan–nanocrystalline cellulose	Mixed matrix membrane	Nano-silica and NaCl	-	Increase in hydrophilicity, 98% rejection of produced water	Produced water treatment	[169]

**Table 4:** Recent studies on wastewater remediation using several biopolymeric nanocomposites as adsorbents.

Nanocomposite	Pollutant	Adsorption Equilibrium Time (min)	Isotherm Model and Kinetics	Removal Efficiency/ Adsorption Capacity	Refs.
GO/polyamidoamine	Pb (II), Cd (II), Cu (II), Mn (II)	60	Langmuir and pseudo-second-order	568.18, 253.81, 68.68, 18.29 mg/g	[171]
Chitosan/silica/ZnO	Methylene blue	-	Langmuir and pseudo-second-order	293.3 mg/g	[85]
Molecularly imprinted polymer (MIP) chitosan-TIO_2_	Rose Bengal	-	Langmuir and pseudo-second-order	79.365 mg/g	[174]
PAMAM–titaniananohybrid	Phenol	-	Langmuir and pseudo-second-order model	77 mg/g	[175]
PPI dendrimers functionalized with long aliphatic chains	Fluoranthene, phenanthrene, pyrene	-	-	19, 67, 57 (mg/g)	[176]
Chitosan-MnO_2_	Cr (VI)	120	Langmuir and intra-diffusion	61.56 mg/g	[189]
NTiO_2_-chitosan@NZrO_2_-chitosan	Gd (III) Sm (III)	30 20	Langmuir–Freundlich and pseudo-first-order	450 650 μmol/g	[190]
Chitoson-MoS_2_	Cr (IV) U (VI) Eu (III)	180 120 240	Langmuir	3.05 0.71 0.86 mmol/g	[191]
Chitosan-benzil/zinc oxide/Fe_3_O_4_	Remazol brilliant blue	-	Freundlich and pseudo-second-order	620.5 mg/g	[192]
Chitosan-PVA@CuO	Acidblue 25	-	Langmuir and pseudo-second-order	171.4 mg/g	[193]
Chitosan/zero-valent iron	Direct red 81	-	Freundlich and pseudo-first-order	61.35 mg/g	[194]
ZnO/chitosan nanocomposite	Congo red	-	Langmuir	227.3 mg/g	[195]
Chitosan-ZnO	Malachite green	-	Langmuir and pseudo-second-order	11 mg/g	[196]
Chitosan–silica	Methyl orange	-	Langmuir	7 mg/g	[197]
Chitosan/SiO_2_/CNTs	Direct blue 71 (DB71) Reactive blue 19 (RB19)	-	Langmuir and pseudo-second-order	61.35 mg/g 97.08 mg/g	[198]
Polyacrylonitrile/PAMAM composite nanofibers	Direct red 80, Direct red 23	-	Langmuir and pseudo-second-order kinetics	2000 mg/g	[199]
GO-PPI dendrimer	Acid red 14, Acid blue 92	-	Langmuir and pseudo-second-order kinetics	434.78, 196.08 mg/g	[200]
Chitosan-Cu/Al@N-C microspheres	Oxytetracycline antibiotics	-	Langmuir and pseudo-second-order kinetics	92.25%, 1727.65 mg/g (25 °C)	[177]
O-carboxymethyl chitosan (O-CMC)/oxidized pectin hydrogel-EDTA acid-LDH	Benzylpenicillin	-	Langmuir and pseudo-second-order kinetics	250 mg/L	[201]
